# A Retrospective Cohort Study of the Association of Inpatient Amlodipine Dose With Renal Complication Rates and Hospital Length of Stay

**DOI:** 10.7759/cureus.46237

**Published:** 2023-09-29

**Authors:** Eric R Gottlieb, Stephen S Gottlieb

**Affiliations:** 1 Hospital Medicine, Mount Auburn Hospital, Cambridge, USA; 2 Medicine, Harvard Medical School, Boston, USA; 3 Institute for Medical Engineering and Science, Massachusetts Institute of Technology, Cambridge, USA; 4 Cardiology, University of Maryland School of Medicine, Baltimore, USA

**Keywords:** amlodipine, hypertension, hospital length of stay, acute kidney injury, medication reconciliation

## Abstract

Background

Correct hospital medication reconciliation is important for continuity of care, but optimal home antihypertensive medication ordering has not been adequately studied. Since excessive hospital blood pressure control is associated with adverse renal and cardiovascular outcomes, we assessed the association of inpatient doses of amlodipine (10mg vs. 5mg) with length of stay and renal failure and fluid and electrolyte disorders (RF/FED).

Methods

In this retrospective cohort study, clinical and demographic data on patients not initially admitted to the ICU between 2008 and 2019 were extracted from the Medical Information Mart for Intensive Care (MIMIC-IV). Multivariable logistic regression was used to assess the association between amlodipine dose during the first 24 hours of admission and RF/FED. Multivariable linear regression was used to assess the association between amlodipine dose and length of stay when controlling for RF/FED or maximum blood urea nitrogen (BUN) concentration and other confounders.

Results

There were 5,932 patients included in this study, and 3,038 of whom received 10mg of amlodipine. A 10mg dose of amlodipine was associated with an increased likelihood of RF/FED (OR: 1.248, 95% CI (1.104, 1.412), p<0.001). It was also associated with a longer length of stay (coef.: 0.338, 95% CI (0.067, 0.609), p=0.015). This was not significant when controlling for RF/FED (dose coef.: 0.197, 95% CI (-0.070, 0.464), p=0.147) or maximum BUN (dose coef.: 0.082, 95% CI (-0.147, 0.312), p=0.482).

Interpretation

Higher amlodipine dose was associated with longer length of stay, and this is likely mediated by RF/FED. Randomized trials are needed to determine which home blood pressure medications should be ordered in the hospital.

## Introduction

Approximately 50% of Americans over 20 years of age have hypertension, with a higher prevalence in older patients and those with comorbidities such as diabetes and chronic kidney disease [1]. When patients with hypertension are hospitalized, complete medication reconciliation is important. Absent any clear contraindications, many providers will order most or all of the patients’ regular home medications, including their antihypertensives.

During acute illness, however, patients’ blood pressures may be lower or more variable than they are at home due to many factors, including poor oral intake and other differences in diet, volume, sepsis, adrenal insufficiency, and anemia or hemorrhage [2]. Patients may also present to the hospital with an overly intensive outpatient regimen due to “white coat hypertension” affecting clinic blood pressure readings [3] or may not be taking their medications as prescribed. Hypertension management is often further complicated by elevations in blood pressure in the emergency department due to pain and anxiety, although associations between elevated emergency department blood pressures and long-term hypertension have been observed [4].

Several recent studies have shown that inpatient escalation of blood pressure regimens and administration of intravenous and other “as-needed” antihypertensive medications are associated with higher rates of adverse events, including acute kidney injury, stroke, and myocardial infarction [5,6]. However, optimal management of home blood pressure medications during hospital admissions has not been adequately studied.

Amlodipine is a commonly used long-acting dihydropyridine (DHP) calcium channel blocker that has been available since the 1980s with simple once-daily dosing [7]. It is considered safe for most patients, and its pharmacokinetics is not significantly affected by renal impairment [8]. It is therefore an attractive prototype to study the impact of routine inpatient blood pressure management decisions on clinical outcomes, such as adverse renal events and hospital length of stay.

Because patients not receiving amlodipine would be expected to have important differences in the prevalence of hypertension and/or clinical conditions at the time of hospital admission, there are limitations to comparing cohorts of patients who did and did not receive the drug. In contrast, the dose of amlodipine, while related to blood pressure, is probably impacted significantly by physician preference and other factors not associated with the severity of the disease. For these reasons, we compared hospital lengths of stay in patients who received the two most common daily doses of amlodipine, 5mg and 10mg, respectively, to assess dose effects while minimizing and controlling for potential confounders.

## Materials and methods

Research ethics

This retrospective cohort study is based on the Medical Information Mart for Intensive Care (MIMIC-IV), which includes patients who were admitted to the Beth Israel Deaconess Medical Center (BIDMC) in Boston, MA, between 2008 and 2019 [9]. MIMIC was approved for research by the institutional review boards of BIDMC (2001-P-001699/14) and the Massachusetts Institute of Technology (0403000206) without a requirement for individual patient informed consent because it is deidentified and publicly available.

Cohort selection

MIMIC-IV and MIMIC-IV-ED for emergency department data were queried from the Google BigQuery (Alphabet Inc.) cloud platform using RStudio Version 2022.07.2 (RStudio PBC) with the R 4.0.3 programming language (R Foundation for Statistical Computing). While MIMIC-IV is primarily a critical care database, it includes hospital-level data for both intensive care unit (ICU) and non-ICU patients, including electronic medication administration records (eMAR), laboratory values, and International Classification of Diseases (ICD) codes.

Amlodipine administration records were extracted from eMAR-derived tables. Aggregate amlodipine dose for the first 24 hours after hospital admission, which would likely represent a continuation of the daily home dose, was calculated for each patient. Patients were then filtered to include only those who received aggregate doses of either 5mg or 10mg, and only the first of these hospital admissions for each patient was included. Patients were excluded if their total hospital length of stay was greater than 60 days, due to outliers such as prolonged “social”/placement admissions, if creatinine and blood urea nitrogen (BUN) values during the first 24 hours of admission were not available, or if ICD codes were unavailable. Other data points were collected, including demographic data, discharge disposition, laboratory values, and administration of angiotensin-converting enzyme inhibitors (ACEis) and angiotensin receptor blockers (ARBs). Hospital length of stay was calculated as the number of days between documented hospital admission and discharge. This study followed the Strengthening the Reporting of Observational Studies in Epidemiology (STROBE) guidelines for observational studies [10].

Determination of Elixhauser scores

International Classification of Diseases (ICD) revisions 9 and 10 codes were extracted. For each hospital admission, the Elixhauser comorbidity score was calculated [11] using the R "comorbidity" [12] package. This score aggregates ICD codes into a set of 30 binary diagnostic categories according to the previously published criteria [13].

Elixhauser diagnoses of renal failure (RF) and fluid and electrolyte disorders (FED) were combined into a single binary composite variable, which is abbreviated as “RF/FED.” This composite was positive if either or both of these diagnoses were positive. Given that the potential impact of inpatient management on renal failure and fluid and electrolyte disorders was examined in this study, adjusted Elixhauser scores were also calculated with the renal failure and fluid and electrolyte disorder categories excluded to give an unbiased measure of baseline comorbidity burden. As a negative control, a second composite outcome was created, which included depression and deficiency anemia (DEPRE/DANE), both of which would be unlikely to be precipitated by antihypertensive medication.

Statistical analysis

Categorical variables were compared using a Fisher’s exact test. A logistic regression was performed for the outcome of RF/FED as a function of amlodipine dose, controlling for covariates including age and admission BUN and creatinine. In this model, the coefficient for the categorical variable for dose represents the odds ratio for RF/FED if the 10mg dose was given.

Multivariable linear regression was used to assess length of stay as a function of amlodipine dose and other covariates and to determine whether renal failure and fluid and electrolyte disorders mediated this effect. For categorical variables (10mg dose as compared with 5mg dose, RF/FED or DEPRE/DANE diagnoses, and ACEi/ARB administration), the coefficients and 95% confidence intervals indicate the additional length of stay predicted if the variable is positive.

Linear regression models were developed as follows: First, the association between length of stay and amlodipine dose was assessed when controlling for admission creatinine and BUN concentrations, age, and coadministration of ACEi or ARBs (binary) during the first 24 hours after admission. Gender was excluded for nonsignificance.

The same analysis was then performed with a subset excluding patients with RF/FED, and conversely, including only patients with RF/FED. This was based on the hypothesis that an increased proportion of patients with adverse outcomes, which may be associated with longer length of stay, would prolong the average length of stay for the full cohort, but an effect would not be seen within each individual stratum of renal failure and/or fluid and electrolyte disorders.

A linear regression for length of stay was then performed with the full cohort, controlling for the RF/FED diagnosis. For an alternative measure of these adverse effects, in another model, RF/FED was replaced by the maximum BUN concentration during the admission; maximum creatinine was not significant in the preliminary analysis and was excluded. If these adverse events mediated the longer length of stay, there would not be a significant effect of amlodipine when controlling for them. As a negative control, discussed in the text only, a linear regression was performed controlling for DEPRE/DANE, which would not be expected to be significantly affected by amlodipine dose. In all models, nonsignificant variables were excluded, except where noted. A p-value <0.05 was considered statistically significant.

## Results

Cohort characteristics

There were 5,932 patients included in this study, 2,894 of whom received 5mg of amlodipine and 3,038 of whom received 10mg. Of all the patients who received 5mg or 10mg in the first 24 hours, a total of 1,071 patients were excluded (610 of whom received 5mg of amlodipine and 461 of whom received 10mg). There were eight patients who were excluded due to hospital length of stay greater than 60 days (5mg: 4, 10mg: 4) and 1,062 due to missing admission BUN or creatinine values in the first 24 hours (5mg: 606, 10mg: 456). There was one patient (10mg) who was excluded for both criteria. Patients in the 5mg group received amlodipine on a median (IQR) of 3 (1, 5) distinct days, and patients in the 10mg group received amlodipine on 3 (2, 5) distinct days. Patient characteristics, including demographics and clinical variables stratified by amlodipine dose, are shown in Table 1.

**Table 1:** Clinical and demographic characteristics of subjects stratified by amlodipine dose. Median (interquartile range) values are shown for non-normally distributed continuous variables, and mean (standard deviation) values are shown for normally distributed variables. RF/FED: renal failure/fluid and electrolyte disorders, ACEi/ARB: angiotensin-converting enzyme inhibitor/angiotensin receptor blocker, BUN: blood urea nitrogen, ED: emergency department, DEPRE/DANE: depression/deficiency anemia.

Variable	Combined	5mg Amlodipine	10mg Amlodipine
N	5,932	2,894	3,038
Mean Age in Years (SD)	66.6 (14.1)	68.2 (14.1)	65.0 (14.0)
Male (%)	3,249 (54.8)	1,535 (53.0)	1,714 (56.4)
Ethnicity (%)			
White	3,601 (60.7)	1,927 (66.6)	1,674 (55.1)
Asian	212 (3.6)	114 (3.9)	98 (3.2)
Black	1,363 (23.0)	506 (17.5)	857 (28.2)
Hispanic	308 (5.2)	141 (4.9)	167 (5.5)
Other	448 (7.6)	206 (7.1)	242 (8.0)
Insurance (%)			
Medicaid	319 (5.4)	140 (4.8)	179 (5.9)
Medicare	2,882 (48.6)	1,495 (51.7)	1,387 (45.7)
Other insurance	2,731 (46.0)	1,259 (43.5)	1,472 (48.5)
Died (%)	38 (0.6)	18 (0.6)	20 (0.7)
Mean Elixhauser Score (SD)	4.2 (2.0)	4.0 (2.0)	4.3 (2.0)
Mean Elixhauser Score Without RF/FED (SD)	3.5 (1.7)	3.5 (1.7)	3.6 (1.7)
ACEi/ARB (%)	2,098 (35.4)	949 (32.8)	1,149 (37.8)
Median Sodium (mEq/L) (IQR)	139.0 (137.0, 142.0)	140.0 (137.0, 142.0)	139.0 (137.0, 142.0)
Median Potassium (mEq/L) (IQR)	4.1 (3.8, 4.5)	4.1 (3.8, 4.5)	4.1 (3.8, 4.5)
Median Chloride (mEq/L) (IQR)	102.0 (99.0, 105.0)	102.0 (100.0, 105.0)	102.0 (99.0, 105.0)
Median Bicarbonate (mEq/L) (IQR)	25.0 (22.0, 27.0)	25.0 (23.0, 27.0)	25.0 (22.0, 27.0)
Median BUN (mg/dL) (IQR)	19.0 (14.0, 30.2)	18.0 (13.0, 28.0)	20.0 (14.0, 33.0)
Median Creatinine (mg/dL) (IQR)	1.1 (0.8, 1.6)	1.0 (0.8, 1.4)	1.1 (0.8, 1.8)
Median Maximum BUN (mg/dL) (IQR)	23.0 (16.0, 37.0)	22.0 (15.0, 33.0)	25.0 (16.0, 41.0)
Median Maximum Creatinine (g/dL) (IQR)	1.2 (0.9, 1.8)	1.1 (0.8, 1.6)	1.2 (0.9, 2.1)
Median White Blood Cells (K/µL) (IQR)	7.7 (5.9, 10.0)	7.7 (5.9, 10.0)	7.7 (5.9, 10.0)
Median Hemoglobin (g/dL) (IQR)	11.3 (9.7, 12.7)	11.4 (9.9, 12.8)	11.1 (9.5, 12.7)
Median Platelets (K/µL) (IQR)	209.0 (164.0, 263.0)	208.0 (162.0, 260.0)	210.0 (167.0, 264.8)
Median ED Triage Systolic BP (mmHg) (IQR)	146.0 (130.0, 162.0)	145.0 (129.0, 161.0)	147.0 (130.0, 162.0)
Median ED Triage Diastolic BP (mmHg) (IQR)	76.0 (65.0, 87.0)	76.0 (66.0, 87.0)	76.0 (65.0, 87.0)
First Care Unit (%)			
Medicine	1,766 (29.8)	850 (29.4)	916 (30.2)
Medicine/Cardiology	606 (10.2)	282 (9.7)	324 (10.7)
Other Care Unit	3,560 (60.0)	1,762 (60.9)	1,798 (59.2)
RF/FED (%)	2,994 (50.5)	1,318 (45.5)	1,676 (55.2)
DEPRE/DANE Control (%)	1400 (23.6)	675 (23.3)	725 (23.9)
Mean Length of Stay in Days (SD)	5.0 (5.3)	4.8 (5.0)	5.1 (5.5)
Median Total Amlodipine Days (IQR)	3.0 (2.0, 5.0)	3.0 (1.0, 5.0)	3.0 (2.0, 5.0)

Patients receiving 10mg of amlodipine were more likely to be Black, were younger, and were more likely to be male. They were also more likely to be administered an ACEi or ARB during the first day. They had slightly higher systolic blood pressures at emergency department triage (~28% missing values for systolic blood pressure).

Association between amlodipine dose and renal outcomes

In the 10mg group, 55.2% of patients were classified as having renal failure or a fluid and electrolyte disorder (RF/FED) based on the ICD codes reported for the admission, as compared with 45.5% in the 5mg group, giving an odds ratio for this outcome of 1.47 (95% CI 1.33, 1.63) (p<0.001). In-hospital mortality rates in the 10mg group (0.7%) and 5mg group (0.6%) were not significantly different (OR: 1.06, (95% CI 0.53, 2.13), p=0.87).

In logistic regression, a 10mg dose of amlodipine was associated with an increased likelihood of the RF/FED composite diagnosis (OR: 1.248, 95% CI (1.104, 1.412), p<0.001), when controlling for admission BUN (OR: 1.022, 95% CI (1.014, 1.031), p<0.001), creatinine (OR 5.974, 95% CI (4.898, 7.323), p<0.001), and age (per year OR: 1.015, 95% CI (1.010, 1.020), p<0.001). Administration of ACEi or ARB was not associated with the likelihood of RF/FED in this cohort when controlling for other covariates (OR: 0.923, 95% CI (0.813, 1.046), p=0.209).

Association between amlodipine dose and length of stay

**Figure 1:**
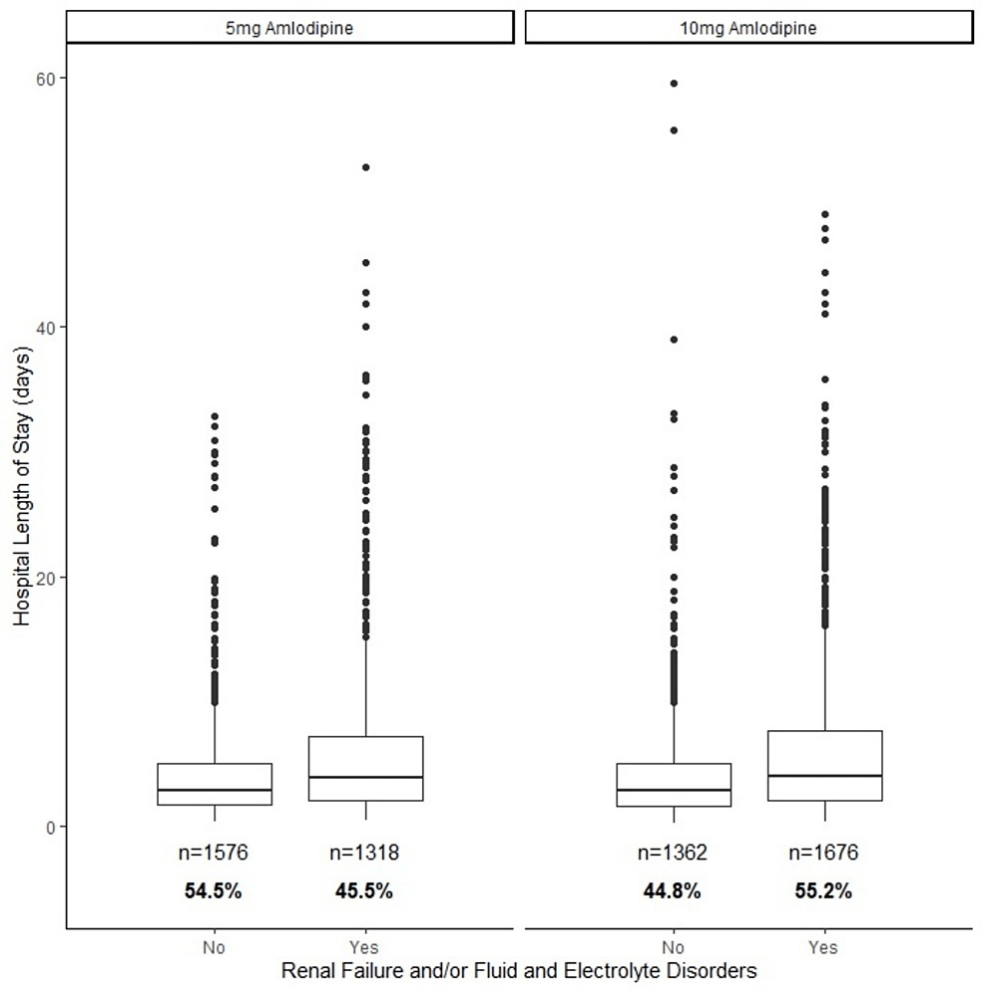
Hospital length of stay for all patients, stratified by amlodipine dose in the first 24 hours and Elixhauser diagnosis of renal failure and/or fluid and electrolyte disorders (RF/FED). In the 10mg cohort, a larger proportion of patients were positive for the RF/FED composite outcome and these patients had longer hospital lengths of stay. Median and quartiles are shown.

Figure 1 shows hospital length of stay for patients who received 5mg and 10mg of amlodipine in the first 24 hours, respectively, stratified by Elixhauser diagnosis of renal failure and/or fluid and electrolyte disorders. Patients who received 10mg of amlodipine had a mean (SD) length of stay of 5.1 (5.5) days, compared with 4.8 (5.0) days in the 5mg group.

As shown in Table 2, in a multivariable linear regression, a 10mg amlodipine dose was associated with a longer length of stay than a 5mg dose (coef.: 0.338, 95% CI (0.067, 0.609), p=0.015), when controlling for age, initial creatinine, initial BUN, and administration of an ACEi or ARB during the first 24 hours of admission. Amlodipine dose was not associated with a significant difference in length of stay when excluding patients with renal failure and/or fluid and electrolyte disorders (coef.: 0.160, 95% CI (-0.136, 0.455), p=0.289), or conversely, when only including patients with these diagnoses (coef.: 0.255, 95% CI (-0.190, 0.700), p=0.261).

**Table 2:** Linear regression models of associations between amlodipine dose and length of stay. Coefficients (95% CI) for categorical variables indicate the additional predicted length of stay if positive. For all analyses, a p-value <0.05 was considered statistically significant. LOS: length of stay, ACEi/ARB: angiotensin-converting enzyme inhibitor/angiotensin receptor blocker, BUN: blood urea nitrogen, RF/FED: renal failure/fluid and electrolyte disorders.

Variable	LOS	LOS, No RF/FED	LOS, RF/FED Only	LOS Controlling for RF/FED	LOS Controlling for Maximum BUN
Amlodipine 10mg	0.338 (0.067, 0.609)	0.160 (-0.136, 0.455)	0.255 (-0.190, 0.700)	0.197 (-0.070, 0.464)	0.082 (-0.147, 0.312)
	p=0.015	p=0.289	p=0.261	p=0.147	p=0.482
Age (years)	0.016 (0.006, 0.025)				
	p=0.002				
ACEi/ARB first 24 hours	-0.860 (-1.144, -0.576)	-0.702 (-1.003, -0.402)	-0.862 (-1.346, -0.379)	-0.766 (-1.046, -0.485)	-0.819 (-1.060, -0.577)
	p<0.001	p<0.001	p<0.001	p<0.001	p<0.001
Admission BUN (mg/dL)	0.028 (0.019, 0.037)	0.041 (0.018, 0.064)	0.012 (0.001, 0.024)	0.016 (0.007, 0.025)	-0.243 (-0.256, -0.229)
	p<0.001	p<0.001	p=0.037	p=0.001	p<0.001
Admission Creatinine (mg/dL)	-0.103 (-0.195, -0.011)	-0.442 (-0.971, 0.087)	-0.165 (-0.271,-0.058)	-0.182 (-0.270, -0.094)	-0.208 (-0.283, -0.132)
	p=0.027	p=0.101	p=0.002	p<0.001	p<0.001
RF/FED				1.838 (1.544, 2.132)	
				p<0.001	
Maximum BUN (mg/dL)					0.266 (0.255, 0.277)
					p<0.001
N	5,932	2,938	2,994	5,932	5,932

There was not a significant association between amlodipine dose and hospital length of stay when controlling for the RF/FED diagnosis (coef.: 0.197, 95% CI (-0.070, 0.464), p=0.147), which was itself associated with longer length of stay (coef.: 1.838, 95% CI (1.544, 2.132), p<0.001). There was also not a significant association between amlodipine dose and hospital length of stay (coef.: 0.082, 95% CI (-0.147, 0.312), p=0.482) when controlling for maximum BUN (coef.: 0.266, 95% CI (0.255, 0.277), p<0.001).

Finally, we controlled for a composite of depression and deficiency anemia (DEPRE/DANE), which was also associated with longer length of stay (coef.: 0.490, 95% CI (0.174, 0.805), p=0.002) but would not be expected to mediate the effect of amlodipine on length of stay like RF/FED might. In this model, there was a significant association between a higher amlodipine dose and longer length of stay (coef.: 0.337, 95% CI (0.066, 0.608), p=0.015) with a coefficient similar to the first model presented (coef.: 0.338, 95% CI (0.067, 0.609), p=0.015). Other covariates were similar as well (age coef.: 0.017, 95% CI (0.007, 0.026), p=0.001; ACE/ARB coef.: -0.862, 95% CI (-1.145, -0.578), p<0.001; admission BUN coef.: 0.027, 95% CI (0.018, 0.037), p<0.001; admission creatinine coef. -0.098, 95% CI (-0.190, -0.006), p=0.036).

## Discussion

In this study, we showed that a higher dose of amlodipine (10mg) as compared with a lower dose (5mg) in the first 24 hours of hospitalization was associated with a longer hospital length of stay. This effect was abrogated when controlling for maximum BUN or administrative diagnoses of renal failure and/or fluid and electrolyte disorders, and in subsets with and without these diagnoses, all of which suggest that the incidence of renal failure and/or fluid and electrolyte disorders may mediate the observed effect. By contrast, when controlling for an alternative adverse outcome, which was also associated with more severe illness and longer hospital length of stay but not physiologically affected by amlodipine, the effect of amlodipine remained significant. Additionally, we showed in logistic regression that a higher dose of amlodipine is associated with an increased likelihood of renal failure and/or fluid and electrolyte disorders, again controlling for age and admission BUN and creatinine.

It has been previously shown that in hospitalized patients with non-cardiovascular admission diagnoses, intensification of blood pressure treatment, including as-needed (PRN) hypertension treatment, is associated with adverse outcomes such as acute kidney injury, myocardial injury, stroke, and increased hospital length of stay [5,6,14]. Our findings suggest that in certain patients, routine ordering of oral antihypertensive medication such as amlodipine may have similar adverse effects. Given that acute kidney injury and fluid and electrolyte disorders have previously been shown to be associated with adverse outcomes including length of stay, substantially increased cost, and mortality [15-17], it is logical that this adverse effect of amlodipine would impact length of stay as well. Although the Elixhauser diagnoses of renal failure and fluid and electrolyte disorders may not distinguish between preexisting and incident events, we controlled for admission BUN and creatinine to effectively isolate the marginal effect.

Amlodipine was chosen for this study because of the simple dosing regimen, with most patients taking either 5mg or 10mg once daily, providing a straightforward comparison. It is also not known to have specific acute kidney injury or electrolyte risk factors comparable to those of ACE inhibitors and angiotensin receptor blockers (ARBs) [18], and therefore, cases of significant hypotension notwithstanding, ordering decisions are more likely to be dictated by individual physician practice, rather than patient-specific factors. We also used a dose-effect strategy to minimize potentially strong confounders such as baseline blood pressure, comorbidities, current illness severity, and other antihypertensive medications, which we would likely encounter if we compared patients who did and did not receive amlodipine.

This evidence is consistent with trials of longer-term blood pressure control, such as the landmark SPRINT trial, which showed that even with superior outcomes with intensive blood pressure control, adverse events including hypotension, electrolyte abnormalities, and acute kidney injury were more common with intensive treatment [19]. However, given that most benefits of blood pressure control accrue over the long term, while acute hypotension can cause end-organ injury, including acute kidney injury, stroke, and myocardial infarction, the short-term benefits of intensive blood pressure control in the hospital may not justify the risks of ordering some or all of these medications in this setting.

A notable secondary finding was that in all length of stay models, administration of an ACE inhibitor or ARB was associated with a significantly shorter hospital length of stay and it was not associated with the likelihood of RF/FED. This may either be due to a confounding effect of illness severity, whereby sicker patients would be less likely to receive these medications due to the known risks of acute kidney injury and hyperkalemia, or it could indicate that the known renal and cardioprotective effects of these medications are significant in the inpatient setting. The latter hypothesis would be supported by prior studies showing that when calcium channel blockers such as amlodipine are combined with ACE inhibitors or ARBs, there is a reduced risk of peripheral edema and potentially lower cardiovascular risk [20].

The strengths of this study are that it included a large number of diverse patients and that high-resolution data were used to evaluate the same outcome in several different ways with consistent results. Limitations include that it was from a single center, it was retrospective, and the determination of adverse events based on ICD codes and limited laboratory values may not provide a full clinical picture. There are potential confounding factors, such as the possibility that differences in the severity of hypertension between patients receiving 5mg and 10mg of amlodipine led to differences in outcomes. A randomized controlled trial would be needed to fully address these concerns, although other retrospective techniques such as propensity scoring may also be of value. That said, the observed differences shown in Table 1 were small and would be variably expected to increase or decrease the risk of adverse outcomes. Additionally, both cohorts were large, diverse, and similar in size, and we controlled for other relevant factors including age, renal function at admission, and administration of ACEi and ARBs. Further research is needed to confirm these findings and determine whether they extend to other common blood pressure medications. Medication reconciliation is necessary to understand the patient, but it may be risky to assume that all medications should be continued in the hospital.

## Conclusions

Administering a 10mg dose of amlodipine during the first day of hospital admission, which likely represents the continuation of the home antihypertensive dose, is associated with a longer hospital length of stay than a 5mg dose. This is likely mediated by increased incidence of renal failure and fluid and electrolyte disorders. Our findings are consistent with prior observational studies showing negative outcomes associated with more intensive inpatient blood pressure control, but further research is needed for verification, ideally by way of a randomized controlled trial.
